# The effect of a single session of 30-min mindful breathing in reducing fatigue among patients with haematological cancer – a randomised controlled trial

**DOI:** 10.1186/s12904-021-00855-7

**Published:** 2021-10-15

**Authors:** Diana-Leh-Ching Ng, Gin-Gin Gan, Nur Adila Anuar, Yu-Zhen Tung, Natalie-Zi Lai, Yi-Wen Tan, Siti Norazilah Mohd Said, Amalia Madihie, Chee-Shee Chai, Seng-Beng Tan

**Affiliations:** 1 Department of Medicine, Faculty of Medicine and Health Science, University Malaysia Sarawak, Kota Samarahan, Sarawak Malaysia; 2 Department of Medicine, Faculty of Medicine, University of Malaya, Kuala Lumpur, Malaysia; 3 Faculty of Cognitive Sciences and Human Development, University Malaysia Sarawak, Kota Samarahan, Sarawak Malaysia

**Keywords:** Haematological cancer, Fatigue, Mindful breathing, Edmonton symptom assessment system, Functional assessment of chronic illness therapy fatigue scale

## Abstract

**Background:**

Patients with haematological cancer had considerable symptom burden, in which fatigue was the most prevalent. Almost 70% of haematological cancer patients reported fatigue.

**Methods:**

We conducted a parallel-group, non-blinded, randomised control trial at the haemato-oncology unit of University Malaya Medical Centre, from 1st October 2019 to 31st May 2020. Patients included were ≥ 18 years, had histopathological diagnosis of haematological cancer, and fatigue score of ≥4 based on the fatigue subscale of Edmonton Symptom Assessment System (ESAS). Patients allocated to the intervention group received standard care plus a guided 30-min mindful breathing session, while those in control group received standard care. The study outcomes include fatigue severity according to the fatigue subscale of ESAS, visual analogue scale of 0 – 10, and Functional Assessment of Chronic Illness Therapy Fatigue Scale Version 4, at minute 0 and minute 30.

**Results:**

Of 197 patients screened, 80 were eligible and they were equally randomised into 30-min mindful breathing versus standard care. Lymphoma (58.9%) was the commonest haematological malignancy, followed by multiple myeloma (13.8%), acute leukaemia (11.3%), myeloproliferative neoplasm (6.3%), chronic leukaemia (5.0%) and myelodysplastic syndrome (5.0%). There was no difference in the demographic and clinical characteristics between the 2 groups.

At minute 0, both arms of patients had similar ESAS-fatigue score (median, 5) and FACIT-fatigue score (mean ± SD, 24.7 ± 10.6 for intervention group versus 24.7 ± 9.7 for control group). At minute 30, intervention group had lower ESAS-fatigue score (median, 3 versus 5) and FACIT-fatigue score (mean ± SD, 17.1 ± 10.5 versus 24.8 ± 11.3) compared to control group. Both the ESAS-fatigue score reduction (median, − 2 versus 0, *p* = 0.002) and FACIT-fatigue score reduction (mean ± SD, − 6.7 versus + 0.8; *p* < 0.001) for the intervention group were statistically significant. The calculated effect size Cohen’s d was 1.4 for between-group comparison of differences in total FACIT-fatigue score.

**Conclusions:**

Our results provide evidence that a single session of 30-min mindful breathing was effective in reducing fatigue in haematological cancer patients. On top of all the currently available methods, 30-min mindful breathing can prove a valuable addition.

**Trial registration:**

NCT 05029024, date of registration 15th August 2021. (Retrospectively registered).

## Background

Haematological cancers include leukaemia, lymphoma, multiple myeloma, myeloproliferative neoplasms and myelodysplastic syndrome [1, 2]. They accounted for 10% of all malignancies and 9.5% of malignancies-related mortality [3]. Patients with haematological cancers have considerable symptom burden, with an average of 8.8 symptoms per patient according to one cross-sectional study [4]. Among the symptoms, fatigue was the most prevalent. Almost 70% of haematological cancer patients reported fatigue.

The National Comprehensive Cancer Network defined cancer-related fatigue (CRF) as a distressing, persistent and subjective sense of physical, emotional, or cognitive tiredness or exhaustion associated with cancer or cancer-related treatment, that is disproportional to recent activity, and interferes with usual functioning [5]. Fatigue was not only the most prevalent symptom of haematological cancer [6–10], it was also the commonest side-effect of haematological cancer treatments such as cytotoxic chemotherapy or marrow suppressive agents [11]. Fatigue might persist for many years or remain for life in patients who had successfully achieved haematological cancer remission, post cytotoxic chemotherapy or haematopoeitic stem-cell transplantation [12–15]. CRF had significant negative impact on patients’ quality of life, daily activities, employment, social relationships and mood [16]

Management of CRF in patients with haematological cancer remained challenging despite studies reporting non-pharmacological measures, particularly exercise and psychological interventions might significantly improve CRF [17, 18]. First, exercise was contraindicated in patients with anaemia, thrombocytopenia, active infection, bone lesion and risk of falls [19]. Second, attending psychological interventions such as cognitive-behavioural therapy, psycho-educational therapy, supportive-expressive therapy and mindfulness-based stress reduction therapy in outpatient setting could be tiring, as well as time-consuming and expensive because these interventions are delivered in multiple sessions over months. Third, pharmacological treatments such as methylphenidate, modafinil and corticosteroid in CRF are still lacking strong evidence, and are not without side-effects [18].

Mindfulness involves paying attention on purpose, in the present moment and non-reactively [20]. It has been shown to reduce fatigue, stress, anxiety, depression and improve sleep [21–25]. However, most evidence on the benefits of mindfulness are based on conventional 6-8 weeks mindfulness programme. Brief mindfulness practices of 5-30 min duration warrant more research. Recent studies have shown that a single session of 20-min mindful breathing significantly reduced dyspnoea in patients with chronic lung disease and decompensated heart failure [26, 27], as well as palliating multiple symptoms in patients with terminal cancer [28]. Brief mindfulness practices require less energy and time from patients, and have the potential advantage of more sustainable habit-formation, when it is practiced regularly. But the evidence for such practices in reducing fatigue is still lacking. In this study, we aim to determine the efficacy of a single session of 30-min mindful breathing in reducing fatigue among patients with haematological cancers.

## Methodology

### Study design

We conducted a parallel-group, non-blinded, randomised control trial at the haemato-oncology unit of University Malaya Medical Centre (UMMC), a tertiary university hospital with 1617 beds in Kuala Lumpur, capital of Malaysia, from 1st October 2019 to 31st May 2020. Patients included were aged 18 years and above, had a histopathological diagnosis of haematological cancer according to World Health Organisation classification, and a fatigue score of ≥4 based on the fatigue subscale of Edmonton Symptom Assessment System (ESAS). Patients were excluded if they had impaired conscious level, cognitive impairment or any psychiatric illness that would prevent them from giving informed consent or participate fully in the study; active or past history of cancer of other system, or a haemoglobin level of < 8 g/dl [29].

### Procedure

Patients with haematological cancer attending the haematology clinic or admitted to the haematology ward of UMMC were consecutively screened for eligibility. The demographic and clinical data of the eligible patients, which include age, gender, ethnicity, religion, occupation, education level, marital status, type of haematological cancer, current status of cancer, types of cancer treatment, duration of cancer, blood parameters and other co-morbidities were obtained from the hospital Electronic Medical Record System. Any missing information was obtained by face-to-face interview with patients or relatives.

Patients who satisfied the inclusion criteria and agreed to participate in the study were randomly assigned into 2 groups based on computer-generated random numbers, in blocks of 10, with a one-to-one allocation ratio. Allocation sequence was concealed with sealed envelopes to prevent selection bias. Patients allocated to the intervention group received standard care plus a guided 30-min mindful breathing session which consisted of four breathing exercises done consecutively in one-to-one manner. The four exercises included identifying the in-and out-breath, following the entire length of the breath, bringing the mind back to the body and relaxing the whole body [30]. Each exercise lasted 7.5 min. Guidance was given by one of the two research assistants, who were medical doctors. They were trained by one of the co-investigators, who was a palliative care physician, certified in mindfulness training.

The training included a brief introduction to the basic concepts of mindfulness, followed by a 30-min mindful breathing session guided by the trainer. Guidance on delivering the intervention with attention to paralanguage (intonation, rate and rhythm of speech, pitch, articulation, use of silence, etc.) and body language (eye contact, facial expression, posture and bodily movement), followed by supervision of the actual delivery of the 30-min mindful breathing session by each research assistant were performed. The instructions for the 30-min mindful breathing are presented in Table 1. Patients in the control group received standard care alone. They were allowed to resume their usual activities 30 min prior to further assessment. Regardless of whether in intervention or control group, patients recruited in out-patient clinic were in sitting position, while patients recruited in ward were in lying position during the study.

**Table 1:** Instructions for 30-min mindful breathing

**Step 1 (7.5 min): Identifying the in-breath and out-breath**	
Make yourself comfortable. Relax your body. Close your eyes gently. Take two deep breaths slowly. Then, breathe naturally. Notice the flow of air through your nose. Rest your attention gently on the breath. Breathing in, you know you are breathing in. Breathing out, you know you are breathing out. In-out, in-out, in-out. If you are distracted by any sounds, body sensations, thoughts or feelings, gently come back to your breath. Be aware of your in-breath and out-breath for the next few minutes.	
**Step 2 (7.5 min): Following the entire length of the breath**	
Continue to relax your body with your eyes closed. Continue to pay attention to your breath. Follow the entire length of your breath. Follow the beginning, the middle and the end of your in-breath, and the beginning, middle and the end of your out-breath. If you are breathing in a long breath, you know you are breathing in a long breath. If you are breathing in a short breath, you know you are breathing in a short breath. If you are breathing out a long breath, you know you are breathing out a long breath. If you are breathing out a short breath, you know you are breathing out a short breath. Do not force yourself to take a long or short breath. Just breathe naturally. Be aware of the entire length of the breath. In-in-in, out-out-out, in-in-in, out-out-out. If you are distracted by any sounds, body sensations, thoughts or feelings, gently come back to your breath. Follow the entire length of your breath for the next few minutes.	
**Step 3 (7.5 min): Bringing the mind back to the body**	
As you follow the entire length of your breath, bring your mind back to your body. Instead of thinking about the past or future, bring your mind back to now. Bring your mind and body together as one. As you breathe in, feel your whole body moving with your breathing in. As you breathe out, feel your whole body moving with your breathing out. Breathing in, you are aware of your whole body as you are breathing in. Breathing out, you are aware of your whole body as you are breathing out. Feel the different parts of your body as you breathe in and out. Then, feel the body as a whole, fully united with your mind. Feel the wholeness of yourself with each breath for the next few minutes.	
**Step 4 (7.5 min): Relaxing the body**	
Once your breathing is harmonious, your body will relax naturally. Feel whether there is any tension in your body. Breathe and relax the tension one by one, from the top to the bottom. Relax your head, face, neck, arms, forearms, hands, chest, abdomen, legs, and feet. Then relax your whole body all at once. Breathing in, you calm your body when you are breathing in. Breathing out, you smile. Again, breathing in, you calm your body when you are breathing in. Breathing out, you smile. In-out-calm-smile, in-out-calm-smile, in-out-calm-smile. Feel your breath flowing through your body and calming your body. Feel your breath leaving your body and smile. Continue to relax your whole body for the next few minutes	

The study outcomes were assessed at minute 0 (before intervention – T0) and minute 30 (after intervention – T30). The outcomes at T0 and T30 include fatigue severity according to the fatigue subscale of ESAS, a unidimensional visual analogue scale (VAS) of 0 – 10, and the score of Functional Assessment of Chronic Illness Therapy (FACIT) Fatigue Scale Version 4, a multidimensional fatigue scale. At the end of the study, patients in the intervention group were asked about their feedback, any harm, and asked if they were satisfied and willing to practise 30-min mindful breathing in their daily life.

The ESAS is a valid and reliable tool to assess nine common symptoms experienced by cancer patients [31]. The nine symptoms assessed include: pain, tiredness, nausea, depression, anxiety, drowsiness, loss of appetite, wellbeing and shortness of breath. An additional blank scale is given to assess each patient’s ‘other problems’ as needed. The severity for each symptom upon assessment has a rating from 0 to 10 on a numerical scale; with 0 indicating absence of the symptom and 10 indicating the worst symptom severity. For this study, the tiredness subscale was chosen to assess participants’ fatigue severity.

The FACIT Fatigue Scale is a 13-item multidimensional assessment tool to measure individual’s fatigue level during their normal daily activities over the past 7 days [32]. It has high internal validity (Cronbach’s alpha = 0.96) and high test-retest reliability (ICC = 0.95). Each participant’s level of fatigue is rated on a five point Likert scale (0 = not at all fatigued to 4 = very much fatigued). The total FACIT-fatigue score ranges from 0 to 52, with a higher score reflecting more fatigue.

The medical ethics approval was obtained from the Medical Ethic Committee of the UMMC (Ethics no: 201971-7588). Written informed consent were obtained from all the participants. The study was conducted according to the Declaration of Helsinki.

### Statistical analysis

The sample size was calculated based on the formula for a randomised control trial for continuous variables with statistical superiority design, n = [2 SD^2^(Z α/2 + Zβ)^2^] divided by d^2^ [33], in which n was sample size in each group; SD was standard deviation from previous study; Z α/2 was desired level of statistical significance, typically 1.96 for type-1 error of 0.05; Zβ was desired power, typically 0.842 for 80% power; d was effect size, the difference in means. In this study, we took the SD of 1.58 and d of 1 [21]. Therefore, the minimum sample size was 78 (39 for each arms).

Results for continuous variables were expressed as mean ± standard deviation (SD), median or inter-quartile range depending on normality of the variable distributions; while results for categorical variables were expressed as percentages. Between-groups differences for continuous data was compared by using independent t-test or Mann-Whitney U test, as applicable; while between-group differences for categorical data was compared by using Chi-Square test or Fisher-Exact test, as applicable. A 2-sided *p*-value of less than 0.05 was considered as significant in this study. Bonferroni correction, 0.05/number of hypothesis was also performed to counteract the problem of multi-testing. Statistical analyses were performed using the software package, Statistical Package for the Social Sciences (SPSS for windows version 25.0, SPSS Inc., Chicago, IL, USA).

## Results

**Fig. 1:**
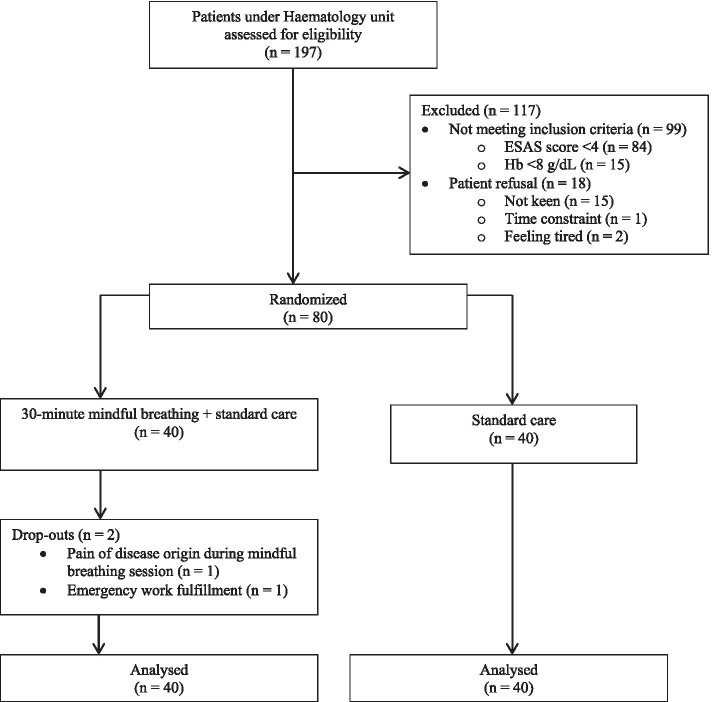
CONSORT diagram: details of enrolment, allocation and analysis

Of 197 patients screened, 80 were eligible and randomised into 30-min mindful breathing (intervention group, *n* = 40) versus standard care (control group, n = 40) (Fig. 1). The patients’ gender was almost equally distributed, with mean age of 54.6 ± 15.4 years (Table 2). Lymphoma (58.9%) was the commonest haematology malignancy, followed by multiple myeloma (13.8%), acute leukaemia (11.3%), myeloproliferative neoplasm (6.3%), chronic leukaemia (5.0%) and myelodysplastic syndrome (5.0%). There was no difference in terms of age, gender, ethnicity, marital status, religion, education level, occupation, type of haematological malignancy, disease status, comorbid or haemoglobin level between the 2 groups.

**Table 2:** Demographic and clinical characteristic of patients with haematological malignancies

Demographic and Clinical Characteristics	Techniques		p-value
	30-min mindful breathing (*n* = 40)	Control (*n* = 40)	
Age, mean ± SD (years)	53.4 ± 16.2	55.8 ± 14.6	0.493^a^
Gender, *n* (%)			
Male	20 (50.0)	19 (47.5)	1.000^b^
Female	20 (50.0)	21 (52.5)	
Ethnicity, *n* (%)			
Malay	21 (52.5)	18 (45.0)	0.792^b^
Chinese	15 (37.5)	17 (42.5)	
Indian	4 (10.0)	5 (12.5)	
Marital Status, *n* (%)			
Single	6 (15.0)	7 (17.5)	0.992^b^
Married	27 (67.5)	26 (65.0)	
Widowed	6 (15.0)	6 (15.0)	
Divorced/Separated	1 (2.5)	1 (2.5)	
Religion, *n* (%)			
Muslim	21 (52.5)	18 (45.0)	0.816^b^
Buddhist	12 (30.0)	13 (32.5)	
Christian	5 (12.5)	5 (12.5)	
Hindu	2 (5.0)	4 (10.0)	
Education Level, *n* (%)			
None	1 (2.5)	1 (2.5)	0.054^b^
Primary	2 (5.0)	11 (27.5)	
Secondary	18 (45.0)	15 (37.5)	
College/ University	19 (47.5)	13 (32.5)	
Occupation, *n* (%)			
None	11 (27.5)	4 (10.0)	0.130^b^
Employed	16 (40.0)	21 (52.5)	
Retired	13 (32.5)	15 (37.5)	
Haematology Malignancy, *n* (%)			
ALL	1 (2.5)	0 (0.0)	0.075^c^
AML	2 (5.0)	6 (15.0)	
CLL	0 (0.0)	1 (2.5)	
CML	3 (7.5)	0 (0.0)	
Lymphoma	24 (60.0)	23 (57.5)	
Multiple myeloma	4 (10.0)	7 (17.5)	
Myelodysplastic syndrome	4 (10.0)	0 (0.0)	
Myeloproliferative disease	2 (5.0)	3 (7.5)	
Remission status, *n* (%)			
Yes	13 (32.5)	11 (27.5)	0.807^b^
No	27 (67.5)	29 (72.5)	
Comorbid, *n* (%)			
No	16 (40.0)	16 (40.0)	1.000^b^
Yes	24 (60.0)	24 (60.0)	
Hb level (g/dL), mean ± SD	11.9 ± 2.3	11.2 ± 2.1	0.144^a^
Baseline ESAS-Fatigue, median (IQR)	5 (2)	5 (2)	0.824^d^
Baseline FACIT-Fatigue, mean ± SD	24.0 ± 10.6	24.0 ± 9.7	0.983^a^

*SD* Standard Deviation, *ALL* Acute Lymphoblastic Leukemia, *AML* Acute Myeloid Leukemia, *CLL* Chronic Lymphocytic Leukemia, *CML* Chronic Myeloid Leukemia, *Hb* Haemoglobin, *ESAS-F* Edmonton Symptom Assessment System-Fatigue component, *FACIT-F* Functional Assessment of Chronic Illness Therapy-Fatigue component, *IQR* Interquartile Range

^a^p-values from Student’s t-test

^b^*p*-values from Chi-square test

^c^p-values from Fisher’s Exact test

^d^p-values from Mann-Whitney U test

At minute 0, both arms of patients had similar ESAS-fatigue score (median, 5) and FACIT-fatigue score (mean ± SD, 24.7 ± 10.6 for intervention group versus 24.7 ± 9.7 for control group) (Table 3). At minute 30, intervention group had lower ESAS-fatigue score (median, 3 versus 5) and FACIT-fatigue score (mean ± SD, 17.1 ± 10.5 versus 24.8 ± 11.3) compared to control group. Both the ESAS-fatigue score reduction (median, − 2 versus 0, *p* = 0.002) and FACIT-fatigue score reduction (mean ± SD, − 6.7 versus + 0.8; *p* < 0.001) for the intervention group were statistically significant (Table 4). The calculated effect size Cohen’s d was 1.4 for between-group comparison of differences in total FACIT-fatigue score.

**Table 3:** Descriptive statistic of ESAS-F and FACIT-F total score

	ESAS-F Score		FACIT-F Total Score	
	30-min mindful breathing, median (IQR)	Control, median (IQR)	30-min mindful breathing, mean ± SD	Control, mean ± SD
Minute 0	5 (2)	5 (2)	24.0 ± 10.6	24.0 ± 9.7
Minute 30	3 (2)	5 (3)	17.1 ± 10.5	24.8 ± 11.3
Score difference	-2 (2)	0 (2)	−6.7 ± 5.9	0.8 ± 4.4

*ESAS-F* Edmonton Symptom Assessment System-Fatigue component, *FACIT-F* Functional Assessment of Chronic Illness Therapy-Fatigue component, *SD* Standard Deviation, *IQR* Interquartile Range

**Table 4:** Changes in ESAS-F and FACIT-F total score: comparison between 30-min mindful breathing and control arm

Outcomes	30-min Mindful Breathing		Control		t or Z	df or U	p-value	d or r
	Median	Mean Rank	Median	Mean Rank	Z	U	Rank Sum Test	r
**ESAS-F score**								
Minute 0	5	39.9	5	41.1	- 0.223	777.5	0.824	
Minute 30	3	33.6	5	45.1	- 2.285	534.5	0.022	
ESAS-F difference	−2	31.6	0	47.0	- 3.065	459.5	0.002	0.12
	Mean	SD	Mean	SD	t	df	t-test	d
**FACIT-F Total score**								
Minute 0	24.0	10.6	24.0	9.7	−0.022	78.0	0.983	
Minute 30	17.1	10.5	24.8	11.3	−3.117	76.0	0.003	
FACIT-F Total difference	−6.7	5.9	0.8	4.4	−6.267	68.4	0.000	1.42

*SD* Standard Deviation, *ESAS-F difference* Difference in ESAS-F score between minute 0 and minute 30, *FACIT-F Total difference* Difference in FACIT-F total score between minute 0 and minute 30

Analysis of FACIT fatigue subscales showed that patients receiving 30-min mindful breathing experienced significant reduction in symptoms 1 (generalised fatigue), 3 (feeling wash-out), 5 (difficulty to initiate work), 6 (difficulty to finish work), and 12 (frustration), compared to standard care patients (all *p* < 0.004) (Table 5).

**Table 5:** FACIT-F individual item score difference: comparison between 30-min mindful breathing and control arm

	Techniques				U	z	p-value	r
	30-min mindful breathing		Control					
	Median	Mean Rank	Median	Mean Rank				
F1 difference**	−1	32.0	0	46.7	473.0	−3.049	0.002*	0.12
F2 difference	−1	35.2	0	43.6	598.0	−1.692	0.091	0.04
F3 difference	0	32.1	0	46.5	480.0	−3.045	0.002*	0.12
F4 difference	−1	33.5	0	45.2	531.5	−2.389	0.017	0.07
F5 difference	−1	31.7	0	46.9	464.5	−3.135	0.002*	0.13
F6 difference	−1	30.6	0	47.9	423.0	−3.586	0.000*	0.17
F7 difference	0	38.8	0	40.2	733.5	−0.287	0.774	0.00
F8 difference	0	36.7	0	42.2	653.0	−1.133	0.257	0.02
F9 difference	0	34.8	0	44.0	581.0	−1.931	0.054	0.05
F10 difference	0	34.7	0	44.1	578.0	−2.067	0.039	0.06
F11 difference	0	36.3	0	42.6	637.5	−1.465	0.143	0.03
F12 difference	0	30.0	0	48.6	397.5	−4.189	0.000*	0.23
F13 difference	0	33.3	0	45.4	524.5	−2.593	0.010	0.09

*p-value < 0.004, (Bonferroni correction, 0.05/13)

**Difference in FACIT-F individual item score between minute 0 and minute 30

Regarding the feedback from patients in the intervention group, the majority of them found the 30-min mindful breathing useful in reducing their fatigue. The mindful breathing script was good. They felt calm and peaceful as they focused on their breathing, and were able to forget their worrying thoughts. Many slept during the session due to their extreme tiredness and felt better on waking up, which was not seen in the control group. Some patients reported that they would continue to practice themselves. A few asked for more guided sessions. Duration wise, some said it could be longer; some said it was too long. No harm was reported by any of the participants in the intervention group.

## Discussion

The results showed that a single session of 30-min mindful breathing was effective in reducing fatigue rapidly in hematological cancer patients. The strength of the fatigue reduction was large with a Cohen’s d effect size of 1.4. To date, two other clinical trials have shown beneficial effects of mindfulness-based interventions on cancer-related fatigue [34, 35]. However, the intervention period of both studies was longer. The first was a 9-week mindfulness-based cognitive therapy (MBCT) [34]. The second was an 8-week mindfulness-based stress reduction (MBSR) [35].

Although conventional mindfulness practice could potentially produce a longer lasting effect on fatigue reduction, a single session of brief mindfulness practice offers an immediate bedside option to palliate fatigue in hematological cancer patients. The script of the guided 30-min mindful breathing is simple and the practice is easy to deliver. The instructors for the brief mindfulness practice need only a single session of mindfulness training. This has the potential of making the intervention easily available to cancer patients.

Other non-pharmacological interventions that improved cancer-related fatigue included home-based walking exercise program and relaxation therapy [36, 37]. As for pharmacological interventions, psychostimulants such as methylphenidate and modafinil, and dexamethasone have shown promise in reducing cancer-related fatigue [38, 39]. Thirty minutes mindful breathing could be a useful complement to the above-mentioned interventions.

Conducting the guided 30-min mindful breathing in the ward setting was not without its challenges. Approaching newly-admitted fatigued patients was difficult because most of them were unwell to undergo the session. Patients who had been admitted for some time or were near hospital discharge were more receptive for participation in the study. Other challenges were interruption from staff for checking vital signs, serving meals or medications, and cleaning. A quiet environment will be more conducive to practice. Few patients expressed difficulty concentrating for 30 min and suggested a shorter session tailored to patient’s energy. For the clinic patients, delivering the session in a private room was commendable. Quantitative feedback from patients pertaining to 30-min mindful breathing should be taken in future study.

The study has several limitations. This is a single center study. We were unable to blind the patients because their active participation was required. There was a lack of an active control. The outcome measures were subjective. We did not include any objective measurement of fatigue. We explored the immediate effect of the intervention and not the sustained effect. Multiple sessions may be necessary to produce a longer lasting effect. Many patients in the intervention arm slept during the session due to their extreme tiredness and felt better on waking up. Therefore, the significant reduction of fatigue among them could be due to the effect of the intervention itself, or the intervention may help the patients felt asleep and subsequently reduce the fatigue. There were also those who requested for audio recordings for home-practice.

## Conclusion

To conclude, our results provide evidence that a single session of 30-min mindful breathing was effective in reducing fatigue in haematological cancer patients. Fatigue, disabling and the most prevalent symptom in hematological malignancies, warrants the development of better methods of management. On top of all the methods, 30-min mindful breathing can prove a valuable addition.

## Data Availability

The datasets used and/or analysed during the current study are available with the corresponding author on reasonable request.

## Abbreviations

CRF: Cancer-related fatigue
UMMC: University Malaya Medical Centre
ESAS: Edmonton Symptom Assessment System
T0: minute 0 - before intervention
T30: minute 30 - after intervention
VAS: Visual analogue scale
FACIT: Functional Assessment of Chronic Illness Therapy
n: sample size in each group
SD: Standard deviation
Z α/2: Desired level of statistical significant
Zβ: Desired power
d: Effect size
SPSS: Statistical Package for the Social Sciences
MBCT: Mindfulness-based cognitive therapy
MBSR: Mindfulness-based stress reduction
ALL: Acute lymphoblastic leukemia
AML: Acute myeloid leukemia
CLL: Chronic lymphocytic leukemia
CML: Chronic myeloid leukemia
Hb: Haemoglobin
ESAS-F: Edmonton Symptom Assessment System-Fatigue component
FACIT-F: Functional Assessment of Chronic Illness Therapy-Fatigue component
IQR: Interquartile Range
